# Infant Feeding Websites and Apps: A Systematic Assessment of Quality and Content

**DOI:** 10.2196/ijmr.4323

**Published:** 2015-09-29

**Authors:** Sarah Taki, Karen J Campbell, Catherine G Russell, Rosalind Elliott, Rachel Laws, Elizabeth Denney-Wilson

**Affiliations:** ^1^ Faculty of Health University of Technology Sydney Sydney Australia; ^2^ Centre for Obesity Management and Prevention Research Excellence in Primary Health Care (COMPaRE-PHC) Sydney Australia; ^3^ Centre for Physical Activity and Nutrition Research Deakin University Melbourne Australia

**Keywords:** applications, Internet, infant feeding, health information, quality, suitability, readability

## Abstract

**Background:**

Internet websites and smartphone apps have become a popular resource to guide parents in their children’s feeding and nutrition. Given the diverse range of websites and apps on infant feeding, the quality of information in these resources should be assessed to identify whether consumers have access to credible and reliable information.

**Objective:**

This systematic analysis provides perspectives on the information available about infant feeding on websites and smartphone apps.

**Methods:**

A systematic analysis was conducted to assess the quality, comprehensibility, suitability, and readability of websites and apps on infant feeding using a developed tool. Google and Bing were used to search for websites from Australia, while the App Store for iOS and Google Play for Android were used to search for apps. Specified key words including baby feeding, breast feeding, formula feeding and introducing solids were used to assess websites and apps addressing feeding advice. Criteria for assessing the accuracy of the content were developed using the Australian Infant Feeding Guidelines.

**Results:**

A total of 600 websites and 2884 apps were screened, and 44 websites and 46 apps met the selection criteria and were analyzed. Most of the websites (26/44) and apps (43/46) were noncommercial, some websites (10/44) and 1 app were commercial and there were 8 government websites; 2 apps had university endorsement. The majority of the websites and apps were rated poor quality. There were two websites that had 100% coverage of information compared to those rated as fair or poor that had low coverage. Two-thirds of the websites (65%) and almost half of the apps (47%) had a readability level above the 8th grade level.

**Conclusions:**

The findings of this unique analysis highlight the potential for website and app developers to merge user requirements with evidence-based content to ensure that information on infant feeding is of high quality. There are currently no apps available to consumers that address a variety of infant feeding topics. To keep up with the rapid turnover of the evolving technology, health professionals need to consider developing an app that will provide consumers with a credible and reliable source of information about infant feeding, using quality assessment tools and evidence-based content.

## Introduction

### Background

The Internet has become a popular medium for consumers seeking health-related information [1]. The proportion of the population regularly accessing the Internet is large and growing: The Australian Bureau of Statistics reports that 83% of Australians were using the Internet in 2012 and 2013 compared to 76% in 2010 [2]. In 2014, the Internet was predominantly accessed via desktop computer (81%) compared with 19% who used mobile phones [3]. However, there was a 33% increase of people using their mobile phone to access the Internet from 2012 to 2013 [4]. Recent data suggest that searching for health and medical information was one of the top 15 reasons for accessing the Internet among Australians over 14 years of age [5]. In addition to websites, smartphone apps represent another increasingly popular source of health information [6]. A recent US consumer survey identified that one fifth of smartphone owners have downloaded a health app [7]. It is estimated that presently there are more than 100,000 health-related apps available and, with the growth of smartphone ownership, the use of health apps will continue to rise [8].

Increasingly, parents are turning to the Internet for information and support on how and what to feed infants and toddlers in different life stages [9] including infant feeding practices such as breastfeeding, formula feeding, introducing solids, and also the type of foods to introduce [10]. A Google Consumers Survey found that expecting parents conduct Internet searches twice as frequently as nonparents [11]. However, there are concerns regarding the quality of information provided on websites and apps about infant feeding as this may lead to the adoption of inappropriate practices [12].

There is evidence to show that many eating habits and preferences are formed in infancy and childhood and carried through to adulthood [13]. Because poor eating habits such as eating too many energy-dense foods or eating too few fruits and vegetables begin in early life, there is a key opportunity to support parents to get healthy eating established in early life [14,15]. Given this, it is important that the information provided to parents is continuously updated and consistent with the latest evidence-based infant and child feeding guidelines, such as the *Infant Feeding Guidelines: Information for Health Workers* available from the Australian government’s National Health and Medical Research Council (NHMRC) [16]. This will ensure that parents have access to sources of information that are credible and of good quality.

Presently, there is little information on the quality of websites and apps accessible in Australia regarding infant feeding practices even though various tools are available for evaluation of the quality of Web-based health information. The evaluation of quality includes assessing the website content, credibility, currency, accuracy, reliability, readability, and design [17,18]. However, there is evidence that website developers rarely use these tools [19]. Several studies have evaluated the content of websites and apps focused on health issues such as asthma, pain self-management, and warfarin intake and suggest that the quality of the information and user-friendliness of these resources varied substantially [20-22]. The suitability of health information is also an important aspect to consider; in addition to predicting the appropriateness of the information in terms of content and literacy demands, this also measures graphics and layout and cultural specificity [23]. While health information is widely available on the Web, many individuals with poor health and low literacy may not find the information usable [24]. An overestimation of consumer ability to comprehend the information provided on the Internet may increase the risk of misunderstanding [25].

### Objectives

Given the importance of health-related information targeting infancy and early childhood, conducting an analysis on infant feeding websites and apps is timely. This work will help identify appropriateness and suggest ways in which quality and usability can be improved. This is important if we are to effectively engage consumers around the uptake of healthy infant feeding practices. The aim of this systematic analysis, conducted between December 2013 and December 2014, was to critically evaluate 4 items: quality, comprehensibility, suitability, and readability of information available about infant feeding on websites and apps.

## Methods

### Stage 1: Website and App Selection

#### Websites

Infant feeding websites were identified using the Internet Explorer browser and Google and Bing search engines; selection was based on the most commonly used terms in Australia [26,27]. The key search terms used for websites included *infant feeding*, *baby feeding*, *breast feeding*, *infant feeding schedule*, *infant formula*, *formula feeding*, *introducing solids*, *introducing baby solids*, *solids and fussy babies,* and *introducing solids schedule*. These key terms were identified as the most frequently used terms by consumers on Google Trends [28]. A study reports that consumers seldom read beyond the first page of search results for online health information [29]; therefore, the first 30 websites in both of the search engines were screened. The screening of the websites was conducted by researcher LW using predefined inclusion and exclusion criteria. The websites were reviewed if they met the criteria. All websites were cross-checked by researcher ST. Any disagreements regarding which websites should be included in the study were discussed until consensus was reached.

#### Apps

Infant feeding apps were identified by performing searches in the digital application distribution platforms for the 2 largest smartphone operating systems: the App Store for iOS (Apple Inc) and Google Play for Android (a Linux-based system currently owned by Google). The search terms were modified slightly for the medium. Revised terms included *infant feeding*, *baby feeding*, *breast feeding*, *formula feeding*, *bottle feeding*, *baby solids*, *baby food,* and *baby weaning*. All of the apps yielded from the key terms were screened for eligibility as neither the App Store nor Google Play sorts the most commonly used apps by the number of downloads. The screening of iOS apps was conducted by researcher LW, and the screening of Android apps was completed by researcher ST, both using predefined inclusion and exclusion criteria. The apps were reviewed if they met the criteria. All apps were cross-checked by researcher ST. Any disagreements regarding the inclusion of apps in the study were discussed until consensus was reached.

Inclusion criteria for selecting websites and apps for this study included being written in the English language, targeted to parents of infants up to 1 year of age, and last updated after 2002. Websites were also restricted to those which originated from Australia so advice could be compared to the NHMRC’s *Infant Feeding Guidelines*. This requirement did not apply to apps, however, as there are limited methods to restrict country of origin in app stores; to be included they needed to provide at least information on the Australian infant feeding guidelines. The websites and apps must include information on at least one of the following topics around healthy milk feeding behaviors (breast, expressed breast milk, formula feeding, frequency, timing, correct preparation, feeding on demand, nonnutritive feeding, repeated exposure, varied exposure, and reducing exposure to unhealthy food/beverages) or healthy solid food feeding behaviors (age of solid introduction, types of food introduced, repeated exposure, reducing exposure to unhealthy food/beverages). Additionally, websites that could not be accessed due to broken/dead links; apps that were not free; and electronic books, YouTube or other videos, audio files, news, podcasts, blogs, and PDF and Word documents were excluded.

### Stage 2: Website and App Evaluation

#### Quality Assessment

##### Websites

Two validated tools, the Health-Related Website Evaluation Form (HRWEF) [17] and the Quality Component Scoring System (QCSS) [18,30], were used to assess the quality of websites, as they each contain different criteria.

The HRWEF tool is currently used by the nongovernmental organization Health On the Net Foundation in their code of conduct (HONcode) [31] to certify the quality of an array of health-based websites. It assesses the quality of websites by evaluating the content, credibility, currency, accuracy, reliability, readability, and design of Web-based health information. The QCSS is a tool previously used for medical website evaluations [30,32]. The assessment criteria for this tool include purpose of the content; disclosure of authors/sponsors; currency; accuracy and reliability; accessibility and interactivity; readability; and graphics/layout of information [33,34]. The scoring systems of the tools are as follows: in the HRWEF a score of not applicable (0), disagree (1), or agree (2) and in the QCSS no information (0), partial information (1), or complete information (2). A final score assessing each item on both of the tools was calculated. Websites were rated as excellent for scores of 90% or higher, adequate for 75-89%, or poor for less than 75% with the HRWEF. With the QCSS tool, they were rated excellent for scores 80% or higher, very good for 70-79%, good for 60-69%, fair for 50-59%, or poor for less than 50%.

##### Apps

To our knowledge there were no published, validated tools available to evaluate the quality of apps. Given this, a quality assessment tool was developed by author ST (see Multimedia Appendix 1). Tools previously developed from other studies [20,21] did not comprehensively address the quality of apps; therefore, the new tool was based on items from the HRWEF tool used for websites [17] and tools used in previous studies [20,21]. The criteria used to measure the quality of apps included the description of the app, information about the developer, design and layout, navigation, interactivity, content and accessibility, and security and connectivity of the app. The scoring system used in this tool was attained from one of the studies in which the app quality tool was developed [21]. The scoring system included 29 items which either agreed (1) or disagreed (0) that the app met the criteria and 12 items that were scored as 3 if 100% of the app met the criteria, 2 if 50-99% of the app met the criteria, 1 if 1-49% of the app met the criteria, or 0 if the app did not meet the criteria at all. The final scoring system used was similar to that of the HRWEF tool [17], where a final score rated each app as excellent for a score of 90% or higher, adequate for 75-89%, or poor for less than 75% (see Multimedia Appendix 1). The QCSS tool was also used to measure the quality of the apps.

#### Comprehensiveness

Comprehensiveness was an item in the quality tools that assessed the accuracy and coverage of the content available on websites and apps. In addition, assessment criteria with 8 topics and 22 subtopics based on the *Infant Feeding Guidelines* [16] (see Multimedia Appendix 2, with scoring system derived from [35]) were developed to evaluate the consistency of the information provided. For each topic, accuracy was scored as either correct (+1), incorrect (−1), or absent (0) in turn measuring the amount of topics covered in each website and app. Completeness, the breadth of information provided on each topic, was measured as complete (2) or partially complete (1). A final score in the quality assessment tool included 3 if 100% of information was covered/accurate, 2 if 50% or more of information was covered/accurate, or 1 if less than 50% of information was covered/accurate.

#### Suitability of Information

The Suitability Assessment of Material (SAM) [23] is a validated instrument, which was used to evaluate the appropriateness of information on the websites and apps for the target audience relating to literacy level, cultural appropriateness, content, and layout. The scoring system used for each item measured included not suitable (0), adequate (1), or superior (2), and each website and app was given a final rating of superior (70-100%), adequate (40-69%), or not suitable (0-39%).

#### Readability

The term “readability” refers to the grade level of written text. Readability is an item that was measured with the website and app quality tools and the SAM instrument. Two readability tools were used to measure the content of websites and apps: the Flesch-Kincaid (F-K) [36] and Simple Measure of Gobbledygook (SMOG) [37]. Calculations for F-K were automatically performed using a readability statistics feature available on Word Professional version 2010 (Microsoft, Redmond, WA, USA) by pasting a block of writing from each website or app on the Word document and the reading ease and grade level were recorded. The same block of writing was pasted on an online SMOG calculator that automatically calculated the SMOG and F-K reading grade levels. The average level of reading of US and Canadian adults is between 7th and 8th grade [38,39]. In Australia, literacy competence is measured using the Adult Literacy and Lifeskills Survey, which uses a ranking scale from level 1 (lowest) to level 5 (highest) [40]. As the tools used to measure readability are American, the reading level of information provided could not be compared against the average reading level of Australians. Both the website and app quality assessment tools use a scoring system of agree (2) if the reading level is 8th grade or lower and disagree (1) if the reading level is 9th grade or higher. For the SAM instrument, the scoring was superior (5th grade or lower), adequate (6th to 8th grade), and not suitable (9th grade or higher).

## Results

### Stage 1: Website and App Selection

**Figure 1:**
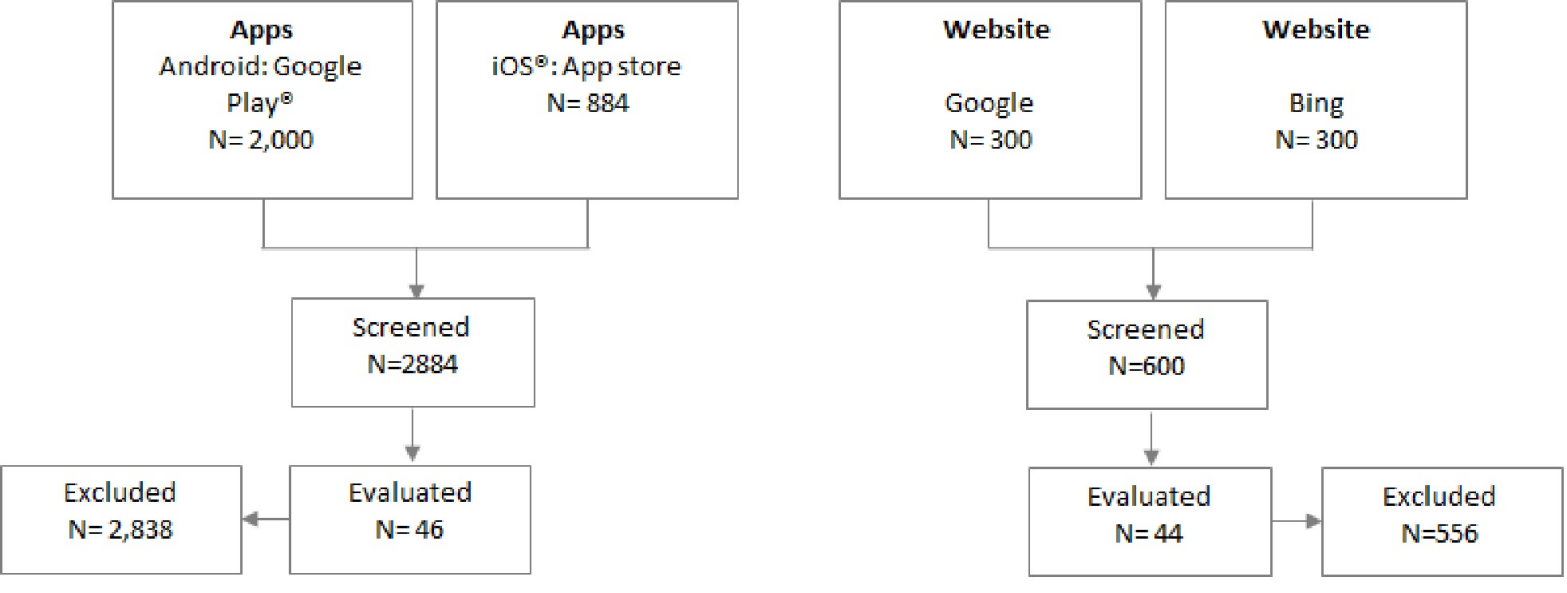
Flow chart of website and app selection.

Searches were performed between December 2013 and March 2014 and rerun in December 2014. In total, 600 websites from Google and Bing and 2884 apps from the app stores for were available for screening (Figure 1). After screening and based on the inclusion criteria, 44 websites and 46 apps were evaluated for the quality, comprehensibility, suitability, and readability of the information. Of the 44 websites, 8 were published by government entities, 10 were sponsored by commercial organizations, and 26 were noncommercial sites from education/nonprofit organizations or hospitals. Of the 46 apps, 2 had university and Australian Breastfeeding Association endorsements, 1 was commercial, and 43 were from noncommercial sites. A numbered list of websites and apps included in this study can be found in Multimedia Appendices 4 and 5, and a summary sheet of the scoring criteria for each evaluation tool can be found in Multimedia Appendix 3.

### Stage 2: Website and App Evaluation

#### Quality Assessment

##### Websites

**Figure 2:**
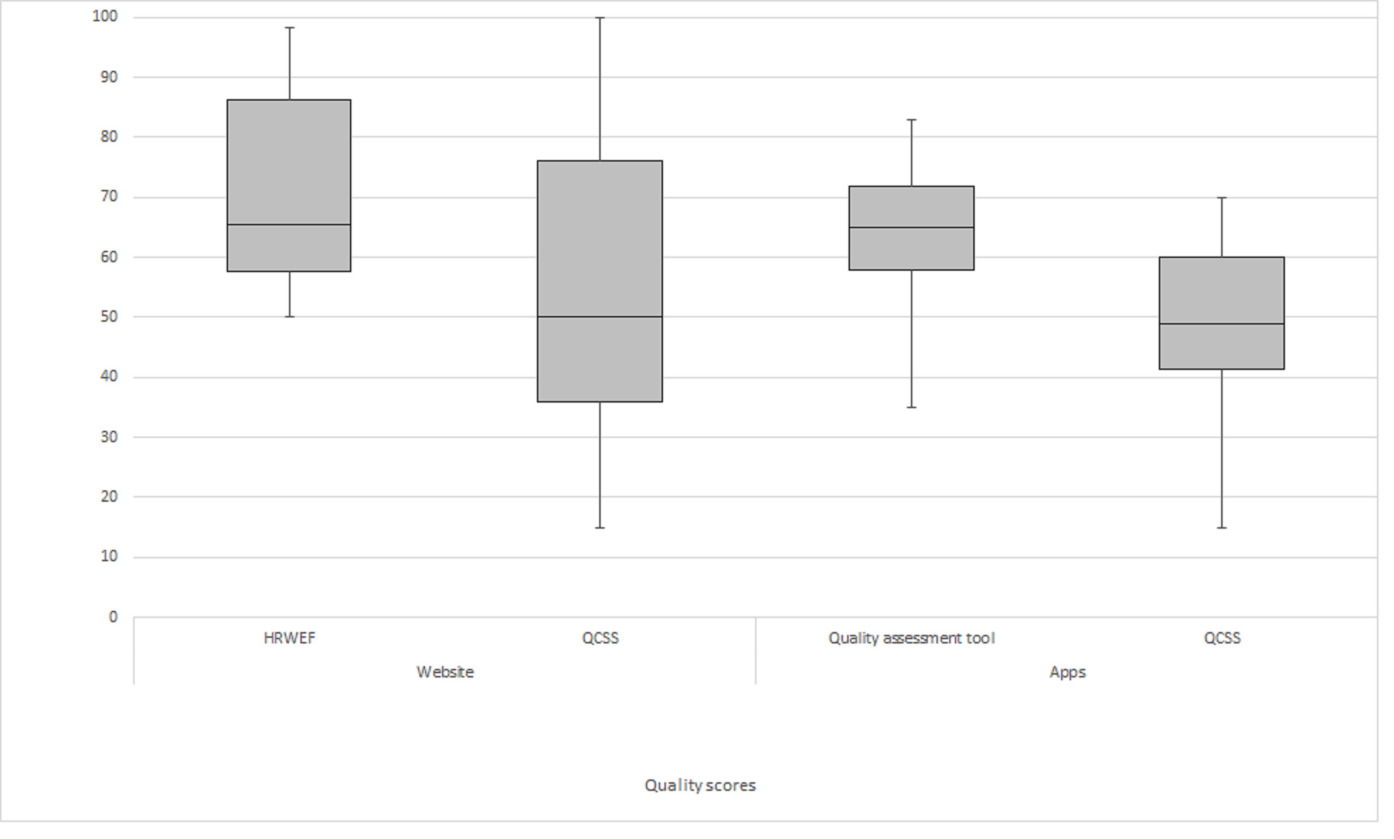
Quality scores of the websites and apps analyzed in this study.

Using the HRWEF tool, the majority of the websites (27/44, 61%) received a poor rating. The median score was determined to be 65% and the interquartile range was 55-86% (Figure 2). Seven of the websites scored an excellent (>90%) rating for quality, and 10 websites received scores of adequate. Four websites stated they subscribed to the HONcode principles.

The QCSS tool revealed that 66% (29/44) of websites were rated poor with a median score of 50% and interquartile range of 36-76%. Two websites were rated excellent, 2 were very good, 7 were good, 4 were fair, and the majority (29/44) was rated poor. Of the 44 websites, 11 reported on author qualifications. Nine of the websites reported that their authors were health care professionals (nutritionists/dieticians, doctors, or nurses/midwives); the authors of 2 websites had no medical expertise (1 was a journalist and 1 was a parent). In regards to the latest content update, 8 websites had not been recently updated to suit the latest infant feeding guidelines (2012) and 7 websites did not identify the date of last update.

Characteristic differences between high- and low-scoring websites varied across the quality items measured. Most websites rated “poor” failed to provide minimal coverage of infant feeding topics, provided inaccurate information, were written at unattainably high reading levels, had not been updated recently, or failed to provide author credentials and external links.

##### Apps

Using the quality assessment tool to measure the quality of apps, 78% (36/46) were rated poor quality, and the median score was 65% with an interquartile range of 58-71% (Figure 2). None of the apps scored excellent, and 10 apps scored adequate. Using the QCSS tool, 91% (42/46) apps were rated poor quality; the median score was 49% with an interquartile range of 41-60%. Four apps were rated fair and 42 were rated poor. Of the 46 apps, 10 reported author qualifications—4 were health professionals (nutritionists/dieticians and nurses) and 6 had no medical expertise. The country of origin for the apps was unidentifiable, but only apps written in American, Australian, and British English were selected. Five apps had not been updated to suit the latest guidelines.

Most apps rated poor had deficits in navigability, design, and color; readability; accessibility (text size and help and search options); and breadth of coverage.

#### Comprehensiveness

##### Websites

Using the *Infant Feeding Guidelines* to assess the comprehensiveness, there were 2 websites that scored 100% for comprehensibility, where all 8 topics about infant feeding (see Multimedia Appendix 2) were included and covered, and the information provided was accurate. Two websites had the lowest comprehensibility score (5%). Inaccurate information about particular infant feeding practices was identified on 2 websites when compared to the guidelines.

##### Apps

Of the 46 apps, the highest score attained for comprehensibility was 78%, and 2 apps scored zero for comprehensibility. Two of the most commonly covered topics in both the websites and apps were Topic 1, encouraging, supporting, and promoting breastfeeding (29/44 and 30/46), and Topic 8, introduction to solids (37/44 and 30/46).

**Figure 3:**
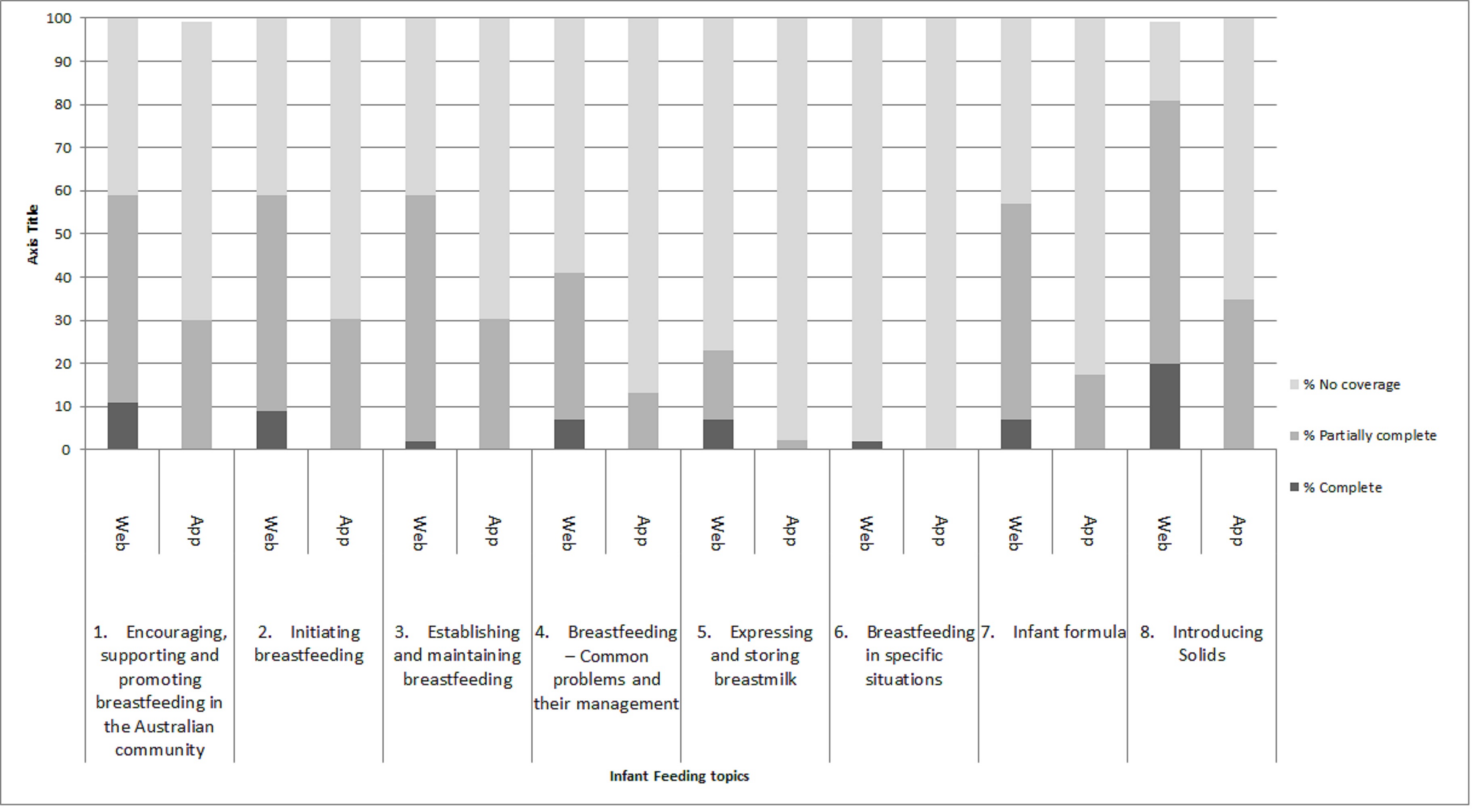
Topics from the Infant Feeding Guidelines provided on websites and apps in this study.

As illustrated in Figure 3, there were very few websites that provided information on all of the subtopics of the infant feeding practices measured in this study. There were no apps that covered the breadth of each topic. Topic 6, breastfeeding in specific situations, was the least covered, with only 2% of websites and no apps covering this topic. Overall, websites covered a wider range of infant feeding topics and provided more extensive information about each topic than the apps, but the completeness of each topic is low.

#### Suitability of Information

##### Websites

Using the SAM tool, 20 websites (45%) received superior rating for suitability, half attained adequate suitability, and 2 (5%) were rated poor. In regards to the individual measures of the SAM criteria identified in Table 1, less than half of the websites addressed learning, stimulation, or motivation. None of the websites or apps addressed cultural specificity of information relating to infant feeding practices from diverse backgrounds and demographics.

##### Apps

The SAM tool was also used to measure the suitability of the apps. There were 7 apps (15%) that achieved superior rating for suitability, 18 apps attained adequate suitability, and 19 (42%) apps were rated poor.

**Table 1:** Infant feeding website and app scores using the SAM criteria.

Characteristic		Websites (n=44) n (%)		Apps (n=46) n (%)	
		Superior^a^	Adequate^a^	Superior^a^	Adequate^a^
**Content**					
	Purpose is evident	34 (77)	10 (23)	20 (43)	8 (17)
	Content about behaviors	43 (98)	—	43 (94)	2 (6)
	Summary and review	3 (7)	7 (16)	—	5 (11)
**Literacy demand**					
	Reading grade level	1 (3)	15 (36)	—	17 (39)
	Writing style, active voice	39 (89)	3 (9)	42 (89)	5 (11)
	Vocabulary uses common words	41 (93)	3 (7)	46 (100)	—
	Context is given first	41 (93)	2 (5)	46 (100)	—
**Graphics**					
	Cover graphic shows purpose	16 (36)	22 (50)	43 (94)	—
	Type of graphics	20 (45)	8 (20)	31 (67)	—
	Relevance of illustrations	29 (66)	3 (7)	23 (50)	5 (11)
	List and tables explained	3 (7)	1 (2)	—	—
	Captions used for graphics	3 (7)	3 (7)	5 (11)	40 (89)
**Layout and typography**					
	Layout factors	44 (100)	—	46 (100)	—
	Typography	44 (100)	—	46 (100)	—
	Subheadings (chunking) used	33 (75)	3 (7)	46 (100)	—
**Learning, stimulation, motivation**					
	Interaction (question-and-answer format) used	—	4 (9)	5 (11)	5 (11)
	Behaviors are modeled and specific	3 (7)	—	—	3 (7)
	Motivation	—	3 (7)	—	—
**Cultural appropriateness**					
	Cultural image and examples	—	—	—	—

^a^Required score for adequate suitability is 40-69%; superior, 70-100%.

#### Readability

##### Websites

Readability grades for all evaluated websites are shown in Table 2. While there was some variability in the actual readability grades attained, the average was consistent across each of the tools used.

The median readability grade for websites was measured as 9 (interquartile range 8-11) using the F-K test in Word and the online F-K calculator. There were 10 websites that were written at approximately 8th grade level or below, which meets the recommended level of written health information.

The median SMOG readability grade level was measured as 10 (interquartile range 7-10). Using the SMOG formula, 16 of the websites were written at approximately 8th grade level or below.

##### Apps

As presented in Table 2, the median readability grade level was 8 (interquartile range 7-10) for apps using the F-K test in Word and the online F-K calculator. There were 14 apps that were written at approximately 8th grade level or below which meets the recommended level of written health information. The median SMOG readability grade levels for apps were measured as 7 (interquartile range 7-8). Using the SMOG formula, 20 of the apps were written at approximately 8th grade level or below.

**Table 2:** Readability scores.

		F-K grade^a^	F-K grade^b^	SMOG grade
**Websites**				
	Median	9	9	10
	Interquartile range	8-11	8-11	7-10
**Apps**				
	Median	8	8	7
	Interquartile range	7-10	7-10	7-8

^a^Flesch-Kincaid test: Word

^b^Flesch-Kincaid test online

## Discussion

### Principal Findings

To our knowledge, this is the first systematic analysis to evaluate websites and smartphone apps providing information on infant feeding practices. This analysis examined the quality standards of information on infant feeding available to users. It also ascertained that there is a need for the development of reliable websites or apps about infant feeding practices that are accessible to health professionals and the general public.

This systematic analysis found that the majority of the websites and apps on infant feeding had poor quality ratings. In contrast, other studies which have evaluated health-related information from websites using similar tools reported adequate ratings for the majority of included websites [22,32]. Another study analyzing apps for the management of obesity using a developed tool rated the majority of apps as fair [41]. One reason resources regarding obesity treatment and infant feeding may be of poorer quality is that a broader group of interested parties, such as journalists and parents, may be involved in website/app development. This would contrast with medical conditions where we might expect expert input and consequent improvement in quality. In turn, this may impact a number of assessed items including credibility of the source, accuracy and coverage of the information, and use of references. Low quality scores were influenced by the number of authors lacking medical backgrounds developing these resources and also the lack of information about author credibility (missing in 75% of the websites and 78% of the apps). Website credibility is one way in which consumers can make a judgment about the quality of information posted on sites [42]. Without this information, consumers may access low quality sites with misleading and inaccurate information.

Commercial websites scored the lowest quality rating, a finding consistent with other studies [34,43]. This finding supports the proposition that commercially motivated sites may set different criteria for information provision and may not represent the existing evidence-based practices [34]. It is of interest that a British qualitative study analyzing maternal accounts of trust regarding healthy eating information sources reported that food manufacturers were the least trusted source for Web-based health information [44]. Regardless, to minimize the risk of consumers accessing websites that may have misleading or inaccurate information, we propose that website developers should use a tool such as HONcode in the early stages of development. Currently in Australia, only medical apps which are used as diagnostic or monitoring tools require approval from the Therapeutic Goods Administration. General health and well-being apps are not regulated [45]. We propose that health apps should also be examined for approval before becoming available to consumers.

### Certifying Health Websites and Apps

Of note, 4 websites stated they subscribed to HONcode principles. Of these, 2 websites attained excellent quality scores. Therefore, using a tool such as HONcode provides a certified endorsement to indicate good quality and encourages website developers to maintain the quality standards of the organization. A qualitative study found that online health information seekers do not commonly evaluate the credibility of sources [46]. Participants lacked the skill to assess website credibility as there was no report of using the About Us section, disclaimer, or disclosure on the websites. The participants’ perceived method to assess credibility was to eyeball the available source, design, and layout of the website, language used, ease of navigation. Given this, using a certified endorsement on websites has the potential to reduce the burden for consumers to search for good quality websites and apps [47].

Another benefit of using a certified endorsement organization to regulate the quality of websites and apps is to ensure that the information shared is constantly updated and in line with appropriate guidelines; more recently updated websites and apps scored higher in quality than those with earlier dates of revision. These findings are similar to a study that assessed smartphone apps around pharmacology education and reported that apps included in their study had not been updated for several years, and the reliability and accuracy of the content were questioned [48]. However, with the rapid growth of apps and constant update of app versions, there is a need to continuously assess and regulate these sources [48]. A study that examined the evolution of asthma-based apps found that the number of apps on asthma more than doubled over 2 years [49]. Although the study’s findings reported no difference in the comprehensiveness of the information available in the newer apps, they did identify improvements in the features offered. Therefore, later versions of apps scored better due to the ease of navigation, updated content, and appropriate layout and graphics. Furthermore, using a certified endorsement may be a useful strategy for policy makers to regulate the information on health websites and apps before allowing it to become available to the public. Another policy innovation might include action by the NHMRC to provide an app with the release of every new *Infant Feeding Guidelines,* which could be made available to parents and health practitioners. This innovation would be potentially powerful as the people responsible for reviewing the evidence could contribute directly to the dissemination strategy (the app) thus reducing any problems in translating evidence into practice.

Another factor contributing to the poor quality of the websites and apps was the level of comprehensibility, including coverage of topics and the completeness of the information on each topic about infant feeding. Our study found that most websites did not cover a range of topics nor did they provide in-depth information about each topic. Similar findings were identified in a study that analyzed online information about dementia, where very few websites covered all topics [50]. Despite the efficiency that has been associated with using the Internet to find health information, websites that lack in information and do not cover a range of topics become a limitation and are no longer a reliable source [51]. Consumers then need to access various websites or apps to obtain information about a particular health subject. Therefore, website and app designers who do not include a range of topics around health information should consider including references that thoroughly cover topics not discussed [50]. In addition to using appropriate specific guidelines and tools to develop good quality websites and apps, they should consider assessing user requirements specific to health conditions and topics in order to meet user needs and expectations [52].

### Adherence to Health Information Best Practice Principles

From the analysis of this study, 3 websites addressed the widest range of topics and attained high completeness scores, as they provided an appropriate level of detail consistent with the Australian *Infant Feeding Guidelines*. Only 4 websites provided incorrect information. These findings are consistent with other studies which have reported on the comprehensiveness of information related to guidelines [20,42]. Incorrect information provided in resources may have serious implications, as the layperson may not be familiar with the *Infant Feeding Guidelines* and might be misguided in the practice of infant feeding.

This study highlights that most of the websites and apps were written at a reading level of 12th grade. This analysis is consistent with other studies [22,53] and is an important finding given that, as previously noted, the average reading level has been reported to be between 7th and 8th grade [38,39]. It is crucial that app and website developers consider literacy levels of the general population as health-related information may be challenging for users with low literacy skills (poorly educated, culturally diverse background) [54]. It is particularly important given those with the least education and lower reading levels may benefit most from well-targeted information, advice, and support.

In our evaluation of the suitability of infant feeding information, we rated the majority of the websites superior or adequate, whereas most of the apps were rated as poor. Using the SAM criteria, poor graphics and low levels of cultural appropriateness were notably deficient. This finding supports a study [53] that reported from a review of Web-based information on osteoporosis that few websites were culturally appropriate. Australia is ethnically diverse, and Internet access is high across all social groups. Given this, culturally appropriate information should be presented across websites and apps [55]. A study evaluating health information on websites about cancer therapy [56] illustrated the difficulty of presenting information to all ethnic backgrounds. As infant feeding practices can vary with different cultural backgrounds (eg, diets, religious beliefs), it is important for website and app developers to consider identifying these aspects in the early stages of development.

### Limitations and Strengths

There are a number of potential limitations of this study that need to be considered. First, the study was limited to evaluating websites and apps written in the English language and websites targeting the Australian population. Therefore, the findings may not be representative of websites and apps written in other languages or from other countries. Another limitation on this point is the fact that this study included only Australian websites while the apps were accepted regardless of the country of origin. Given this, it may have influenced the findings about the comprehensibility and accuracy of the content. There is a potential that the websites may have attained higher comprehensibility scores compared to apps, as the websites would most likely include information from the Australian guidelines compared to the apps. Another limiting factor which may have impacted quality scores of apps is that app development is in its infancy compared to website development. The fact that there is not yet a published quality tool to measure apps enforces the point that there is still much research that needs to be undertaken around health-related apps. Furthermore, Internet and smartphone apps are continuously updated, limiting the likelihood of receiving similar findings using the search terms from this study if it were replicated. To minimize this limitation, the author used Google Trends to identify commonly searched terms around infant feeding practices. Another limitation identified is that the subjective nature of some quality and suitability criteria may impact variability in scoring. Two researchers conducted searches for websites and apps and measured quality and suitability, but only one of the researchers cross-checked the websites and apps. An important strength of this study was the use of 2 different tools to measure the quality and readability of the websites and apps, a method which in turn enabled a comparison of the results.

### Conclusion

It is evident that there are key areas for improvement to increase the utility of information related to infant feeding practices on websites and apps. A majority of websites and apps were of poor quality and had inappropriately high reading levels; few were given a good rating. There were no apps in this study which addressed all of the topics from the Australian *Infant Feeding Guidelines*. Government implementation of policy or certification systems such as HONcode would enable consumers to identify reliable and appropriate information. It would also would ensure that the readability level is appropriate for vulnerable populations. Involving users early in the development of health apps is advised as establishing ways to merge user requirements with evidence-based content to provide high-quality apps.

## Appendix

Multimedia Appendix 1 Quality criteria assessment for smartphone apps.

Multimedia Appendix 2 Information guide sheet for content (accuracy and coverage).

Multimedia Appendix 3 Summary sheet of the scoring criteria for evaluation tools and items measured.

Multimedia Appendix 4 List of websites included in this systematic analysis.

Multimedia Appendix 5 List of apps included in this systematic analysis.

## Abbreviations

F-K: Flesch-Kincaid grade level formula
HONcode: Health on the Net Foundation Code of Conduct
HRWEF: Health-Related Website Evaluation Form
NHMRC: National Health and Medical Research Council
QCSS: Quality Component Scoring System
SAM: Suitability Assessment of Material
SMOG: Simple Measure of Gobbledygook
