# Supplementary material for: Infant Feeding Websites and Apps: A Systematic Assessment of Quality and Content
Source: Interact J Med Res. 2015 Sep 29;4(3):e18. doi: 10.2196/ijmr.4323 (PMC4704960; doi:10.2196/ijmr.4323)
Supplement: Multimedia Appendix 1 [file ijmr_v4i3e18_app1.pdf]

Quality criteria assessment for smartphone apps

Scoring for content/design and usability

Items judged yes/no were scored:

1 point = yes the application meets the criterion
0 points= no, the application does not meet the criterion

Items marked with an asterisks' (*) were scored as:
3 points = 100% of the application meets the criterion 2 points 50% or more of the application meets the criterion
1 point= less than 50% meets the criterion
0 points= the application does not meet the criterion at all

1. **Currency**

a. Name
b. Software/operating system + version
c. Application name
d. Presence of version
e. Download size
f. Date of last update is clearly posted
g. The revision date is recent enough to account for changes in the field
h. Number of downloads
i. Number of rating
j. Average user rating
k. Overall purpose (as claimed by market description)

2. **Author**

a. Developer
b. The application is sponsored by or is associated with an institution or organisation
c. Created by an individual, authors/ editors credentials (educational background, professional affiliations, certifications, past writings) are clearly stated (Y/N)
d. Have they worked with clinicians/ nutritionists/dieticians/nurses/midwives/ paediatricians/ doctors
e. What other applications have they created
f. Contact information (email address and/or phone number) for the author/editor or webmaster is included

3. **Design**

a. Are the colours used logically related (i.e. complementary colours or colours that fit to a particular theme, such as shades of one particular colour)*

b. Is there a consistent colour theme (Y/N)
c. Are the colour choices visually accessible (easy to read with normal or corrected vision*
d. Information should be presented in a way appropriate for the target audience *
e. Standard text size should be readable for normal/corrected vision without adjustment for those who do not know how to adjust screen *
f. Are the font styles easily legible?*
g. Is there a consistent font theme (i.e. one or two fonts used in a logical manner, not a lot of different fonts used inconsistently)? (Y/N)

4. Navigation

a. All pages should be able to be reached in as few clicks as possible -from the main page of the subsection (e.g. a new record section, or result section), it should not take more than three navigational clicks to reach a page) (Y/N)
b. Content should be structurally separate from navigational elements*
c. Clickable items should stylistically indicate that they are clickable*
d. Navigation should be logical and intuitive with signs obvious and not obscured *
e. Here should be a call to action on every page - no dead ends (Y/N)
f. Information can be retrieved in a timely manner (Y/N)
g. A search mechanism is provided (Y/N)

5. Content

a. Details and content should be presented in a natural and logical order*
b. Details or content suit the purpose of the application and are appropriate for target audience*
c. There should be an "about" page identifying the author and application details (Y/N)
d. Resources for content not written by author should be present (Y/N)
e. Resources not written by the author should be reliable - from a credible source, backed up with evidence (Y/N)
f. Application should be regularly updated to keep up with changes in nutrition literature/guidelines - the last update to be according to the latest guideline (Y/N)
g. All aspects of the subject are covered adequately (/21)*
h. The information is accurate (/21)*
i. The reading level is appropriate for the audience (reading grade of 6th to 8th) (Y/N)

6. Accessibility

a. Is the application available in other languages (Y/N)
b. Can text size be altered or is it possible to zoom into the page? (Y/N)
c. Applications should provide an easy way of inputting data, reducing or avoiding need for user to use both hands*
d. Does the application provide a help section/user guide (Y/N)
e. Does the application provide a way of contacting the developers for support (Y/N)

7. Security

a. Users data should be kept private and safe- can the data be encrypted in the event of loss or malfunction system? (Y/N)
b. Can information be backed up/restored in case of loss/malfunction of device (Y/N)
c. Confidentiality: The app publisher clearly states the privacy policy regarding how you treat confidential, private or semi-private information (Y/N)

8. Interactivity and connectivity

a. Can application connect to other devices (via Bluetooth) (Y/N)
b. Can information be sent via email or other alternative? (Y/N)

9. Software Issues

a. Functionality: All functions of the app operates as expected (Y/N)
b. Presentation and user interface: content has spelling and/or layout mistakes, controls are inoperative or inaccessible (Y/N)
c. Crash: The app stopped responding in a timely way to user input (Y/N
