# Supplementary material for: Infant Feeding Websites and Apps: A Systematic Assessment of Quality and Content
Source: Interact J Med Res. 2015 Sep 29;4(3):e18. doi: 10.2196/ijmr.4323 (PMC4704960; doi:10.2196/ijmr.4323)
Supplement: Multimedia Appendix 2 [file ijmr_v4i3e18_app2.pdf]

Appendix 2

Table 4. Information guide sheet for content (Accuracy and coverage)

**Topics of infant feeding practices**<br><br>**Scoring system:** Correct advice (+1), Incorrect advice (-1), Not addressed (0)	
1. Encouraging and supporting breast feeding	Breastfeeding as the physiological norm**
	Protection and promotion of breastfeeding **
2. Initiating breastfeeding **	Breastfeeding education for parents**
	Physiology of breast milk and breastfeeding **
	The first breastfeed**
3. Establishing and maintaining breastfeeding**	Difficulty establishing breastfeeding **
	Factors affecting establishment of breastfeeding **
	Monitoring an infant's progress**
	Maternal nutrition **
**4.** ***Breastfeeding – Common problems and their management*****	Maternal factors affecting breastfeeding **
	Infant factors affecting breastfeeding **
**5.** ***Expressing and storing breast milk***	Expressing breast milk
	Feeding with expressed breast milk
	Storage of expressed breast milk
**6.** ***Breastfeeding in specific situations***	Tobacco, alcohol and other drugs
**7.** ***Infant formula*****	Preparing infant formula**
	Using infant formula **
	Special infant formula **
8. Introducing solids	When should solids foods be introduced **
	What foods should be introduced **
	Foods and beverages most suitable for infants or that should be used with care **
	Healthy foods in the first 12 months (continued exposure and opportunity to sample a wide variety of healthy foods)**

* Scoring was derived from [35]
** Infant Feeding Guidelines [16]
