# Supplementary material for: Infant Feeding Websites and Apps: A Systematic Assessment of Quality and Content
Source: Interact J Med Res. 2015 Sep 29;4(3):e18. doi: 10.2196/ijmr.4323 (PMC4704960; doi:10.2196/ijmr.4323)
Supplement: Multimedia Appendix 3 [file ijmr_v4i3e18_app3.pdf]

**Evaluation tool/item**	**No. of criteria**	**Scoring system**	**Final composite score**
Quality evaluation of information			
Health-Related Website Evaluation Form (HRWEF)	**29**	0= not applicable<br><br>1= Disagree<br><br>2= Agree	**>** 90% = Excellent<br><br>75-89% = Adequate<br><br><75 = Poor
Quality criteria assessment for smartphone apps	**31**	**Items judged yes/no were scored:**<br><br>1 point = yes the application meets the criterion<br><br>0 points= no, the application does not meet the criterion<br><br>**Items marked with an astericks (*) were scored as:**<br><br>3= 100% of the application meets the criterion<br><br>2= 50% or more of the application meets the criterion<br><br>1= less than 50% meets the criterion<br><br>0 = the application does not meet the criterion at all	**>** 90% = Excellent<br><br>75-89% = Adequate<br><br><75 = Poor
Quality Component Scoring System (QCSS)	**20**	0= No information<br><br>1= Partial information<br><br>2= Complete information	> 80%= Excellent<br><br>70-79%= Very good<br><br>60-69%= Good<br><br>50-59%= Fair<br><br><50%= Poor
**Suitability evaluation of information**			
Suitability evaluation of information	**40**	0= Not suitable<br><br>1= Adequate<br><br>2= Superior	70-100%= Superior<br><br>40-69%= Adequate<br><br>0-39% Not suitable

**Items measured for the quality checks**			
**Information content (Coverage)** *(insert in the Quality assessment tools using the scoring systems for website and app)*	**19**		3= 100% of the application meets the criterion<br><br>2= 50% or more of the application meets the criterion<br><br>1= less than 50% meets the criterion<br><br>0 = the application does not meet the criterion at all
**Information content (accuracy)** *(insert in the Quality assessment tools using the scoring systems for website and app)*	**19**	+1= Correct advice<br><br>-1= Incorrect advice<br><br>0= Not addressed	3= 100% of the application meets the criterion<br><br>2= 50% or more of the application meets the criterion<br><br>1= less than 50% meets the criterion<br><br>0 = the application does not meet the criterion at all
**Reading Level**	Reading grade:<br><br><6th- 8th	0= not applicable<br><br>1= Disagree<br><br>2= Agree	
