# Supplementary material for: Infant Feeding Websites and Apps: A Systematic Assessment of Quality and Content
Source: Interact J Med Res. 2015 Sep 29;4(3):e18. doi: 10.2196/ijmr.4323 (PMC4704960; doi:10.2196/ijmr.4323)
Supplement: Multimedia Appendix 4 [file ijmr_v4i3e18_app4.pdf]

Website number	Website name	Website link	Last updated
1	Breastfeeding and introducing solids - Queensland Health	http://access.health.qld.gov.au	2010
2	Formula feeding- information for parents	http://brochures.mater.org.au	Unidentified
3	Infant feeding - Dietitian Association Australia	http://daa.asn.au	2013
4	Breastfeeding - health A-Z - Women and Newborn Health Service	http://kemh.health.wa.gov.au	2013
5	Baby's first foods - Kids Health @ CHW	http://kidshealth.schn.health.nsw.gov.au	2013
6	Top tips for formula feeding your newborn - Parent Exchange	http://parenting.kidspot.com.au	2013
7	Physical activities for young children - Raising Children Network	http://raisingchildren.net.au	2013
8	Introducing solids - Mater Health and Wellness Clinic	http://wellness.mater.org.au	2012
9	ABC Parenting: babies: nutrition	http://www.abc.net.au	2014
10	Aptamil Gold +1 - infant formula - Aptaclub	http://www.aptaclub.com.au	2014
11	Babycenter	http://www.babycenter.com.au/	2013
12	Baby care - weaning - Better Health Channel	http://www.betterhealth.vic.gov.au	2012
13	Breastfeeding your baby	http://www.birth.com.au	2013
14	Starting solids - Bottle babies	http://www.bottlebabies.org	Unidentified
15	Introducing solids to baby - Bubs Baby Shop	http://www.bubs.com.au	2011
16	Introducing solids - Child Health	http://www.childhealth.com.au	Unidentified
17	Introducing solids - Children's Panadol - Australia	http://www.childrenspanadol.com.au	Unidentified
18	Formula feeding - Cradle2Kindy	http://www.cradle2kindy.com.au	2008

19	Foods for babies (solids) - Child and Youth Health	http://www.cyh.com	2013
20	Formula feeding: the basics - Essential Baby	http://www.essentialbaby.com.au	2013
21	Infant feeding & food safety	http://www.foodauthority.nsw.gov.au	2013
22	Baby and toddler food - Fresh For Kids - the best source of fresh	http://www.freshforkids.com.au	2011
23	Breastfeeding - Queensland Health	http://www.health.qld.gov.au	2013
24	Breastfeeding - Healthinsite	http://www.healthinsite.gov.au	2013
25	Healthy kids: introducing solids	http://www.healthykids.nsw.gov.au	2014
26	Heinz for baby	http://www.heinzforbaby.com.au	2014
27	Baby solids - get tips at Huggies.com.au	http://www.huggies.com.au	Unidentified
28	Infant Program - Home	http://www.infantprogram.org/	2012
29	Sweet dreams - solutions for common infant feeding problems	http://www.infanurture.com.au	2014
30	Introducing solid foods - first food ideas	http://www.karimums.com.au	Unidentified
31	Baby's first foods - starting solids - Kate Freeman Nutrition	http://www.katefreemannutrition.com.au	2014
32	How to introduce solids to your baby - Lifestyle YOU	http://www.lifestyle.com.au	Unidentified
33	Introducing solids - Mother & Baby	http://www.motherandbaby.com.au	2014
34	Baby first solid food - Motherhood.com.au	http://www.motherhood.com.au	2009
35	www.mumzone.com.au	http://www.mumzone.com.au	
36	Breast feeding - mydr.com.au	http://www.mydr.com.au	2009
37	Introducing solids to your baby - Natural Therapy Pages	http://www.naturaltherapypages.com.au	2011
38	Introducing solids - Ngala	http://www.ngala.com.au	2009
39	Infant nutrition - Nurtition Australia	http://www.nutritionaustralia.org	2009
40	Introducing solids after 6 months	http://www.parenthub.com.au	2013

	disadvantages - Parenthub		
41	Philips sterilising baby feeding equipment	http://www.philips.com.au	2013
42	Woolworths baby and toddler club - new infant feeding guidelines	https://woolworthsbabyandtoddlerclub.com.au	2014
43	Australian Breastfeeding Association	https://www.breastfeeding.asn.au/	2014
44	Easy 3 step guide to traditional introduction to solids - Nestle Baby	https://www.nestlebaby.com.au	Unidentified
