# Supplementary material for: Infant Feeding Websites and Apps: A Systematic Assessment of Quality and Content
Source: Interact J Med Res. 2015 Sep 29;4(3):e18. doi: 10.2196/ijmr.4323 (PMC4704960; doi:10.2196/ijmr.4323)
Supplement: Multimedia Appendix 5 [file ijmr_v4i3e18_app5.pdf]

App number	App name	App version	Last update
1	Baby – Sprout	2.02 (trial)	2014
2	Baby Care Master +	3.5 (full)	2014
3	Baby Food Recipe	1.2 (full)	2014
4	Baby Weaning Recipes, Planners and Guides	1.3.1 (full)	2014
5	Babyroo - Baby Log for Breastfeeding	1.3.2 (trial)	2014
6	Child Feeding Guide	1.3.3 (full)	2013
7	Feed Safe (from Australia Breastfeeding Association)	1.0.4 (full)	2014
8	My Baby & Me by Phillips AVENT	4.6 (full)	2014
9	My Baby Today - BabyCenter	2.2.1 (full)	2014
10	Sara Chana's Breastfeeding…	1.1 (full)	2014
11	Super Baby Food	1.03 (full)	2011
12	Tess Daly - The Baby Diaries	1.0.2 (full)	2011
13	The Weaning of Life - by Plum!	1.0.2 (full)	2012
14	Today's Parent Milestones	1.3.2 (full)	2014
15	WebMD Baby	2.0 (full)	2014
16	What's Up Baby Feeding	1.0 (full)	2013
17	What to Expect	6.3.1 (full)	2014
18	baby care and baby care	2.3.1 (full)	2014
19	breast feeding	1.4.7 (full)	2014
20	breast feeding (Makeshift labs)	6.0 (full)	2014
21	breast feeding tips and more	1.5 (full)	2014
22	baby feeding	6.73 (full)	2014
23	the breastfeeding guide	1.0.2 (full)	2014
24	infant meal guide	1.1 (full)	2014
25	breastmilk counts	1.1 (full)	2011
26	baby weaning guide	1.0 (full)	2014
27	The breast app ever	1.0 (full)	2014
28	essential baby care guide	1.5 (full)	2013
29	breastfeeding info	1.0 (full)	2011
30	breastfeeding tips and problems	1.0 (full)	2013
31	247 baby	1.0 (full)	2011
32	Baby	1.5 (full)	2014
33	homemade baby food	5 (full)	2013
34	100+ baby food recipe	1.2 (trial)	2013
35	baby food recipes	8.412 (full)	2014
36	baby food and baby food	1.0 (full)	2013
37	savvy breastfeed guide	1.0 (trial)	2014
38	breast start	1.4 (full)	2014
39	breastfeeding tips	1.5 (full)	2014
40	guide to breastfeeding	1.0 (full)	2014
41	breast is best	1.0 (full)	2012
42	baby weaning tips	1.8 (full)	2012
43	guide for new parents	1.0 (full)	2012

44	baby food	1.0 (full)	2013
45	baby diet	1.0 (full)	2012
46	weaning your baby	1.0 (full)	2013
